# Pathogenicity and Metabolites of Endoparasitic Nematophagous Fungus *Drechmeria coniospora* YMF1.01759 against Nematodes

**DOI:** 10.3390/microorganisms9081735

**Published:** 2021-08-14

**Authors:** Juan Wan, Zebao Dai, Keqin Zhang, Guohong Li, Peiji Zhao

**Affiliations:** State Key Laboratory for Conservation and Utilization of Bio-Resources in Yunnan, Key Laboratory for Southwest Microbial Diversity of the Ministry of Education, Yunnan University, Kunming 650091, China; wanjuan202440@163.com (J.W.); yyg0517@163.com (Z.D.); kqzhang1@ynu.edu.cn (K.Z.)

**Keywords:** endoparasitic nematophagous fungi, *Drechmeria coniospora*, *Meloidogyne incognita*, metabolites, nematicidal, egg hatching

## Abstract

Plant parasitic nematodes cause severe damage to crops. Endoparasitic nematophagous fungi (ENF) are a type of important biocontrol fungi, which can cause disease or kill nematodes by producing various spores. As a major ENF, *Drechmeria coniospora* displays certain potential for controlling plant-parasitic nematodes. In this study, the pathogenicity and secondary metabolites of the endoparasitic fungus *D. coniospora* YMF1.01759 were investigated. The strain *D. coniospora* YMF1.01759 had high infection efficiency against nematodes. The process of infecting nematodes by the strain was observed under an electron microscope. Here, 13 metabolites including one new compound 4(*S*)-butoxy-3-(butoxymethyl)-2-hydroxycyclopent-2-en-1-one (**2**) were isolated and identified from the fermentation products of *D. coniospora* YMF1.01759 cultured in a SDAY solid medium. Furthermore, a bioassay showed that 5-hydroxymethylfuran-2-carboxylic acid (**1**) is toxic to the root knot nematode *Meloidogyne incognita* and affects the hatching of its egg. Thereby, the nematicidal mortality attained 81.50% at 100 μg/mL for 48 h. Furthermore, egg hatching was inhibited at the tested concentrations, compared with water control eggs. This is the first report on the secondary metabolites of the ENF *D. coniospora*. The results indicated that *D. coniospora* could infect nematodes by spores and produce active metabolites to kill nematodes. The biological control potential of *D. coniospora* against nematodes was expounded further.

## 1. Introduction

Plant parasitic nematodes cause severe damage to crops. Nematode disease is a global phenomenon. The annual economic losses owing to nematode infection of crop production are estimated at $173 billion [1]. These are parasitic to a variety of crops such as cucumber, melon, pepper, tomato and fruit, and infect the entire plant including the roots, stems, leaves, flowers, fruit and seeds [2,3]. This scenario demands further research to identify effective albeit environment-friendly alternatives to replace legislatively withdrawn highly toxic nematicides [4,5,6].

Biological control agents such as nematophagous fungi may be a solution when applied in the context of integrated pest management systems. Endoparasitic fungi are a group of nematophagous fungi that produce a variety of special spores to infect free nematodes. Either the spores are swallowed by nematodes and then infect, or these infect by adhering to nematode epidermis [7,8,9]. *Drechmeria coniospora* is an obligate parasitic fungus belonging to the family of Clavicipitaceae. It forms spores that adhere to the cuticle of a range of different nematodes and infects a variety of nematode species [7,10,11,12]. The endoparasitic nematode fungus *D. coniospora* is highly aggressive to nematodes. In greenhouse experiments, it can reduce the number of root-knot nematodes forming galls on tomatoes and alfalfa [9]. 

In general, endoparasitic nematophagous fungi (ENF) infect their hosts using conidia. However, a lot of metabolites including nematicidal compounds were isolated from ENF. A polyketone (phomalactone) was identified from the endoparasitic *Verticillium chlamydosporium* and showed prominently nematicidal activity [13]. Several aurovertins were isolated from *Pochonia chlamydosporia*, and Aurovertins F and D displayed toxicity to the free-living nematode *Panagrellus redivivus* [14]. A series of indole diterpenoids were obtained from the endophytic fungus *Drechmeria* sp., and Drechmerins B and I displayed antimicrobial activity [15,16,17]. According to the genomic data, *D. coniospora* could produce abundant secondary metabolites [11,18]. The species has not been investigated with regard to metabolites. In the present study, the infecting process of *D. coniospora* YMF1.01759 against nematode was observed using scanning electron microscopy. Moreover, a chemical investigation was performed on the strain *D. coniospora* YMF1.01759. As a result, 13 metabolites including one new compound were isolated. In addition, a bioassay showed that 5-hydroxymethylfuran-2-carboxylic acid displayed prominent nematicidal activity and could affect the egg hatching of *M. incognita*. This implies that *D. coniospora* YMF1.01759 can infect nematodes by producing active metabolites.

## 2. Materials and Methods

### 2.1. Materials and Normal Culture

*D. coniospora* YMF1.01759 was deposited in the culture collection of the Key Laboratory for Conservation and Utilization of Bio-resource, and Key Laboratory for Microbial Resources of the Ministry of Education, Yunnan University.

*Meloidogyne incognita*: *M. incognita* was cultured on susceptible tomatoes (*Solanum lycopersicum*) for 40 days under greenhouse conditions (25 ± 3 °C). Infested tomatoes were uprooted, and roots showing galls and egg masses were washed to remove soil. Egg masses were selected and placed on a plastic plate. Then, adequate sterile water was added to cover the egg mass to permit the eggs to hatch. Finally, the second-stage juveniles were available for hatching at 28 °C for five days [2]. 

*Caenorhabditis elegans*: *C. elegans* were transferred onto a freshly prepared nematode growth medium (NGM) agar plate with *Escherichia coli* strain OP50 spread over the surface as a food source. *C. elegans* were obtained by gentle washing from the NGM plate with M9 buffer after culturing at 20 °C for three days.

### 2.2. General Experimental Instruments

The optical rotations were measured with a Jasco DIP-370 digital polarimeter (JASCO Corporation, Tokyo, Japan). UV spectra were recorded on a Shimadzu UV-2401 PC spectrophotometer (Kyoto, Japan). NMR spectra were recorded on Avance III-600 spectrometers (Bruker Biospin, Rheinstetten, Germany) with tetramethylsilane (TMS) as an internal standard. The ESI-MS and HR-ESI-MS were recorded on a Thermo high resolution Q Exactive focus mass spectrometer (Thermo, Bremen, Germany). Column chromatography was performed on silica gel G (200–300 mesh, Qingdao Marine Chemical Inc., Qingdao, China) and Sephadex LH-20 (Amersham Pharmacia). Precoated silica gel GF254 plates (Qingdao Marine Chemical Inc., Qingdao, China) were used for thin layer chromatography (TLC). HPLC was performed on an LC3000 (Beijing Chuangxintongheng Science and Technology Co., Ltd., Beijing, China). Abamectin was purchased as a positive control from Solarbio. 

### 2.3. Assaying of Nematicidal Activity

#### 2.3.1. Pathogenicity of *D. coniospora* YMF1.01759 against *C. elegans*

*D. coniospora* YMF1.01759 was cultured on a PDA plate at 25 °C for 14 days to observe the endoparasitic experiment. Subsequently, approximately 150–200 *C. elegans* were introduced to the plates. The strains and nematodes were co-cultured for seven days, and the plates were observed under a microscope every 12 h. 

The endoparasitic phenomenon of the strain on nematodes was also recorded by scanning electron microscopy (SEM). Preparation of sample for SEM: samples of co-incubation for one-seven days were fixated with 4% glutaraldehyde for 30 min; and dehydrated through a graded series of ethanol (30%, 50%, 70%, 80% and 90%; 15 min in each step); and then immersed successively in 100% ethanol two times for 15 min each, in ethanol:isoamyl acetate (1:1, *v*/*v*) liquor for 10 min and in 100% isoamyl acetate for 10 min. The samples were then transferred to a critical point dryer using liquefied carbon dioxide as transitional fluid.

#### 2.3.2. Nematicidal Activity of Extracts of *D. coniospora* YMF1.01759 on Different Media

Five types of solid media [LB (10.0 g tryptone, 5.0 g yeast extract, 10.0 g NaCl, 18.0 g agar, 1 L water), YMG (4.0 g yeast extract, 20.0 g glucose, 18.0 g agar, 1 L water), PDA (200.0 g potato, 20.0 g glucose, 18.0 g agar, 1 L water), SDAY (10.0 g bacterial peptone, 10.0 g yeast extract, 40.0 g glucose, 18.0 g agar, 1 L water) and RICE (rice 40.0 g, 18.0 g agar, 1 L water)] were used to culture *D. coniospora* YMF1.01759. The culturing was performed at 25 °C for 14 days and was then chopped and extracted with a mixed solvent (EtOAc/MeOH/AcOH, 80:15:5, *v*/*v*/*v*) at room temperature. Those crude extracts were dissolved in methanol for testing the nematicidal activity. The sample (30 μL) was added to a petri dish of diameter 3.5 cm containing 100–150 *M. incognita* and 970 μL distilled water. The final concentrations of the extracts were 5 mg mL^−1^. Distilled water containing 3% MeOH was used as a negative control. There were three replicates for each treatment. All the tested plates were incubated at 28 °C. *M. incognita* juveniles were observed under a light microscope after 24 h, 48 h and 72 h. The nematodes were considered to be dead when these did not move on physically stimulated with a fine needle. The mean percentage mortality was calculated. 

#### 2.3.3. Nematicidal Activity of Compounds

The isolated compounds were tested for the nematicidal activity on the second-stage juveniles of *M. incognita* using MeOH as the solvent. The tested compounds were diluted to different concentrations (400, 200, 100, 50 and 25 μg mL^−1^) to assay the nematicidal activity. Distilled water containing 3% MeOH was used as a negative control. Abamectin (10 μg mL^−1^) was served as a positive control. The compound solution and 100–150 nematodes were added to the 96-well plates. The plates were then placed in a 28 °C incubator. Subsequently, the dead nematodes and total nematodes were counted after 24 h, 48 h and 72 h. The mean percentage mortality was calculated. The nematodes were defined as dead when these showed no movement with physical stimulation and adopted a straight shape. The experiment using pure compound was conducted two times with five replicates. The test method is according to the literature [19]. 

#### 2.3.4. Inhibition of Egg Hatching Activity of *M. incognita*

The compound was tested for inhibition of the egg hatching activity of *M. incognita*. Egg masses were obtained from the infested roots and washed with ddH_2_ O to remove soil. Fresh egg masses were assayed for inhibition of egg hatching activity using 96-well plates. An egg mass was placed in each well, and the compound dissolved in MeOH (the final concentration of MeOH in the test solution was at most 3.0%) was added to the tested wells at 200, 100 and 50 μg mL^−1^. Distilled water containing 3% MeOH was used as a control. The plates containing the various components were incubated at 28 °C. The number of hatched worms was counted under the microscope after one, two and three days. Each treatment was performed two times with three replicates [20].

### 2.4. Extraction and Isolation of Metabolites from D. coniospora YMF1.01759

*D. coniospora* YMF1.01759 was grown on a SDAY solid medium (25 L) at 25 °C for 14 days. The solid fermentation products were cut into small pieces and extracted exhaustively with an EtOAc/MeOH/AcOH mixture solution (80:15:5, *v*/*v*/*v*) five times. The extracts were suspended in water and extracted three times by EtOAc, and *n*-butanol. The EtOAc extract (25.6 g) and *n*-butanol extract (80.4 g) were assayed for nematicidal activity against *M. incognita*. The results showed that the EtOAc extract had a strong nematicidal activity against *M. incognita*. Therefore, the EtOAc extract was used to study the metabolites further.

The EtOAc extract (25.6 g) was fractionated with a silica gel G (200–300 mesh) column eluted with a petroleum ether–EtOAc (50:1 to 0:100) gradient solvent system followed by EtOAc–MeOH (8:1 to 0:100), to yield 13 fractions (Fr.1–Fr.13). Fr.4 (240 mg) was chromatographed on a silica gel column eluted with a CHCl_3_–acetone (100:1 to 1:1) gradient solvent system to yield Fr.4.1–Fr.4.2. Fr.4.2 (14 mg) was separated by Sephadex LH-20 (MeOH) to obtain **11** (4.5 mg). Fr.5 (264 mg) was subjected to semipreparative gradient HPLC [detection wavelength of 254 nm and a mobile phase of MeOH–water (the water reduced from 90% to 50%) at a flow rate of 3 mL min^−1^]. It was then separated by preparational TLC plates (GF_254_) [the sample (7 mg) was dissolved in MeOH and repeatedly subjected to TLC separating plates, followed by CHCl_3_–acetone (10:1)] to yield **2** (4.0 mg). Fr.6 (240 mg) was loaded onto Sephadex LH-20 (MeOH) to obtain three fractions (Fr.6.1–Fr.6.3). Fr.6.1 (124 mg) was subjected to a silica gel column eluted with a petroleum ether–acetone (50:0 to 5:1) gradient solvent system and then purified by Sephadex LH-20 (MeOH) to give **4** (3.7 mg). Fr.6.3 (50 mg) was separated on a column of silica gel (200–300 mesh), eluted with petroleum ether–acetone (50:0 to 5:1) and purified by preparational TLC [the sample (6 mg) was dissolved in CHCl_3_–MeOH (1:1) and repeatedly subjected to TLC separating plates, followed by petroleum ether–EtOAc (4:1)] to yield **7** (4.5 mg). Fr.7 (239 mg) was separated on a Sephadex LH-20 column (MeOH) to give four fractions (Fr.7.1–Fr.7.4). Fr.7.2 (8 mg) was purified by preparational TLC [the sample was dissolved in CHCl_3_–MeOH (1:1) and repeatedly subjected to TLC separating plates, followed by petroleum ether–acetone (3:1)] to yield **5** (3.2 mg). Fr.7.3 (36 mg) was loaded repeatedly on Sephadex LH-20 (MeOH) to yield **6** (4.4 mg). Fr.9 (552 mg) was purified on Sephadex LH-20 (MeOH) to give four fractions (Fr.9.1–Fr.9.4). Fr.9.2 (111 mg) was subjected to semipreparative gradient HPLC [detection wavelength of 254 nm and a mobile phase of MeOH–water (the water reduced from 80% to 40%) at a flow rate of 3 mL min^−1^] and purified by Sephadex LH-20 (MeOH) to obtain **3** (6.3 mg) and **8** (5.2 mg). Fr.9.4 (95 mg) was subjected to a silica gel column eluted with a petroleum ether–ethyl acetate (50:1 to 5:1) gradient solvent system. It was then purified by Sephadex LH-20 (MeOH) to obtain **9** (4.3 mg). Fr.10 (1.0 g) was loaded onto a column of Sephadex LH-20 (MeOH) and then separated by semipreparative gradient HPLC [detection wavelength of 254 nm and a mobile phase of MeOH–water (the water reduced from 90% to 50%) at a flow rate of 3 mL min^−1^]. It was then purified by Sephadex LH-20 (MeOH) to obtain **10** (15.2 mg). Fr.11 (1.748 g) was first chromatographed on a Sephadex LH-20 column (MeOH). It was then subjected to semipreparative gradient HPLC [detection wavelength of 254 nm and a mobile phase of MeOH–water (the water reduced from 95% to 65%) at a flow rate of 3 mL min^−1^]. Subsequently, it was purified by Sephadex LH-20 (MeOH) to obtain **1** (33.5 mg). Fr.12 (1.784 g) was subjected to Sephadex LH-20 (MeOH) to give four fractions (Fr.12.1–Fr.12.4). Fr.12.3 (816 mg) was separated on a column of silica gel (200–300 mesh) and eluted with petroleum ether–ethyl acetate (from 20:1 to 3:1). It was then purified by preparational TLC [the sample (20 mg) was dissolved in MeOH and repeatedly subjected to TLC separating plates, followed by petroleum ether–acetone (5:1)] to yield **12** (3.6 mg). Fr.13 (1.06 g) was loaded on Sephadex LH-20 (CHCl_3_–MeOH, 1:1) to give three fractions (Fr.13.1–Fr.13.3). Fr.13.3 (816 mg) was subjected on a silica gel column (200–300 mesh), eluted with CHCl_3_–MeOH (30:1 to 5:1) and purified by semipreparative gradient HPLC [the mobile phase was MeOH–water (the water reduced from 90% to 50%) at a flow rate of 3 mL min^−1^] to obtain **13** (40.5 mg). 

Physicochemical properties of compounds **1** and **2**:

Compound **1**: white solid; $[\alpha {]}_{D}^{20}$ + 3.9 (c 0.21, MeOH); UV (MeOH) *λ*_max_ (log ε) 197 (3.09), 233 (2.68), 251 (2.80) nm; ^1^ H-NMR (CD_3_ OD, 600 MHz) δ 4.56 (1 H, s, H-6), 6.46 (1 H, d, *J* = 3.4 Hz, H-5), 7.13 (1 H, d, *J* = 3.4 Hz, H-4); ^13^ C-NMR (CD_3_ OD, 150 MHz) δ 162.0 (s, C-1), 145.9 (s, C-2), 119.8 (d, C-3), 110.3 (d, C-4), 160.0 (s, C-5), 57.5 (t, C-6); Negative ESI-MS *m/z* 141 [M − H]^−^; HR-ESI-MS *m/z* 141.01821 [M − H]^−^ (calcd for C_6_ H_5_ O_4_, 141.01824).

Compound **2**: Colorless amorphism; $[\alpha {]}_{D}^{23}$ + 6.17 (c 0.09, MeOH); UV (MeOH) λ_max_ (log ε) 195 (3.40), 212 (3.29), 251 (3.67) nm; ^1^ H-NMR (CD_3_ OD, 600 MHz) and ^13^ C-NMR (CD_3_ OD, 150 MHz) data, see Table 1; positive ESI-MS *m/z* 279 [M + Na]^+^; HR-ESI-MS *m/z* 279.15491 [M + Na]^+^ (calcd for C_14_ H_24_ O_4_ Na, 279.15668). 

### 2.5. Statistical Analysis

Nematicidal activity and egg hatching inhibition assay investigations were performed in two independent experiments. Statistics were obtained by comparing the control performance for each concentration and time. The data were subjected to single-factor ANOVA using SPSS Statistics 17 software. ^a^ *p* < 0.001, ^b^ *p* < 0.01 and ^c^ *p* < 0.05 were considered to be statistically significant.

## 3. Results

### 3.1. Pathogenicity of D. coniospora YMF1.01759 against Nematodes

**Figure 1:**
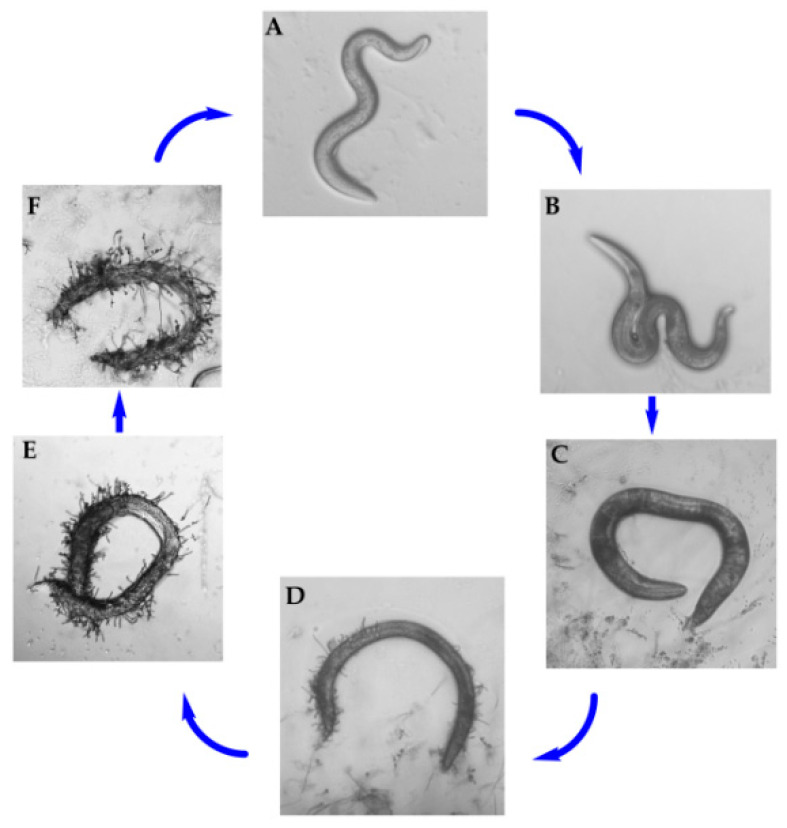
Pathogenicity process of *D. coniospora* YMF1.01759 against *C. elegans*.

In China, the species *D. coniospora* was isolated from soil and first described by Prof. Zhang in our group [21]. The infection cycle of *D. coniospora* on nematodes and the formation of adhesive knobs were observed [11,22]. In our experiment, the pathogenicity of *D. coniospora* YMF1.01759 against the nematodes was first observed under microscope. After *C. elegans* were added to *D. coniospora* YMF1.01759 for 12 h, there was no evident difference in the nematode motility (Figure 1A). At 24 h, the nematode’s activity was weakened significantly, and it displayed a distorted S-shape (Figure 1B). Then, the nematode essentially remained motionless (Figure 1C). After approximately 48 h, the nematode was observed to have sprouted mycelium on the body surface (Figure 1D). Finally, the mycelia spread over the body of the nematodes and produced spores to begin a new infection process (Figure 1E,F). The strain can infect *C. elegans* over various periods. 

**Figure 2:**
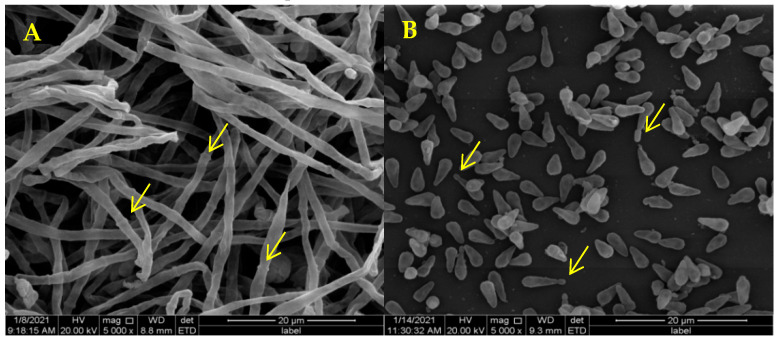
Mycelia (**A**)and Spore (**B**) Morphology of *D. coniospora* YMF1.01759 using SEM.

**Figure 3:**
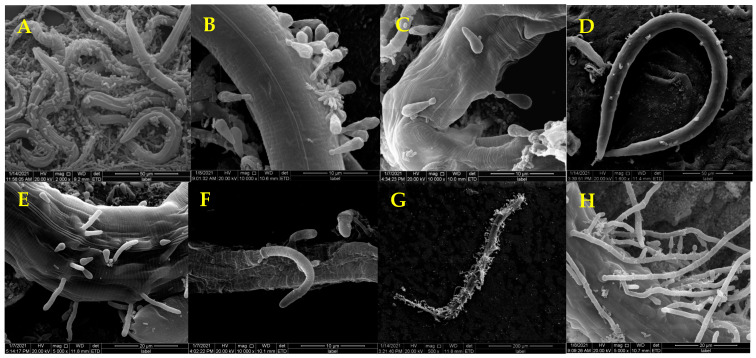
Infection Process of *C. elegans* by Spores of *D. coniospora* YMF1.01759.

SEM showed that the mycelia of *D. coniospora* YMF1.01759 were septate (Figure 2A, yellow arrow) and that the spores were conical (Figure 2B). Adhesive knobs were produced at the tips of the spores when these matured (Figure 2B, yellow arrow). This mainly facilitates the identification of nematodes and adherence to these. When nematodes are added to plates containing *D. coniospora* YMF1.01759, spores produced by the stain simply attach to the nematodes’ body when coming in direct contact. The mature spores gradually adhere to the nematode body wall as the nematodes move (Figure 3A,B), which can adhere to various parts of the nematode body. After the adhesion is completed, the sticky spheres form budding tubes to infiltrate the nematode epidermis (Figure 3C). These then gradually infiltrate the nematode interior, and finally break through the nematode body wall to sprout (Figure 3D–F). Finally, the nematodes decompose (Figure 3G), and mycelia from nematodes produce spores to start a new cycle (Figure 3H). The formation of adhesive knobs is a spontaneous process. It plays a major role in the infestation process [22]. According to a report, chymotrypsin-like proteases are also involved in the infestation process [23]. 

### 3.2. Nematicidal Activity of Medium Screening Extracts

Among the extracts from five media of *D. coniospora* YMF1.01759, the extract from the SDAY medium exhibited strong nematicidal activity against *M. incognita*: 99.1% mortality at a concentration of 5 mg mL^−1^ at 72 h. The other four extracts had no evident activity on *M. incognita* at 5 mg mL^−1^. To track active nematicidal compounds, the crude extract of SDAY was sequentially extracted with EtOAc and *n*-butanol. Two extracts were assayed for nematicidal activity against *M. incognita*. The results showed that the mortality of the EtOAc extracts attained 98.0% at 1 mg mL^−1^ at 72 h. However, the corrected mortality of the *n*-butanol extracts attained 13.3% under identical conditions. Therefore, the EtOAc extract from the SDAY medium was selected to study the active component.

### 3.3. Structure Identification

**Figure 4:**
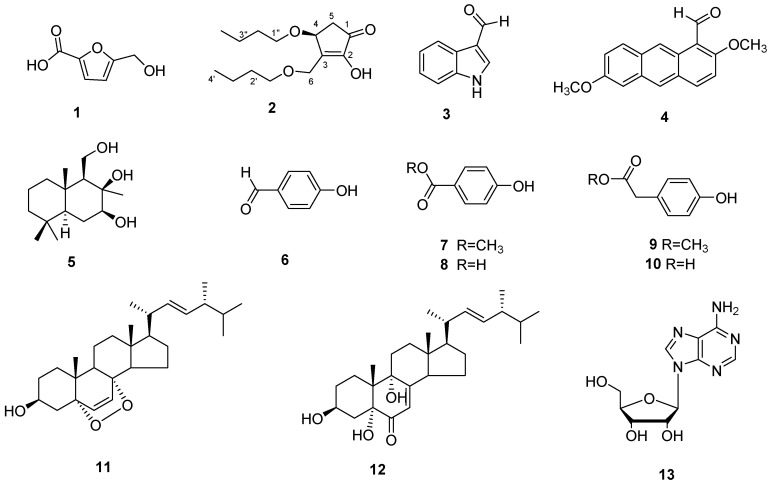
The Metabolites isolated from *D. coniospora* YMF1.01759.

Compound **1** was obtained as a white solid. The HR-ESI-MS data revealed a molecular formula of C_6_ H_5_ O_4_ based on the [M − H]^−^ ion signal at *m/z* 141.01821 [M − H]^−^ (calculated for, 141.01824). ^13^ C-NMR and DEPT spectra indicated that compound **1** contained furan structure (*δ*c 145.9, 119.8, 110.3 and 160.0), a methylene (*δ*c 57.5) and a carboxyl group (*δ*c 162.0). Based on the above data, the structure of compound **1** was identified as 5-hydroxymethylfuran-2-carboxylic acid (Figure 4) [24]. 

**Figure 5:**
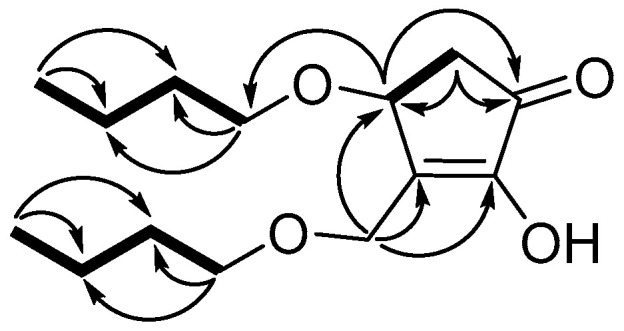
Selected HMBC (arrows) and ^1^ H-^1^ H COSY (bold bond) Correlations of **2.**

Compound **2** was obtained as a colorless amorphism. The HR-ESI-MS data revealed a molecular formula of C_14_ H_24_ O_4_ based on the [M + Na]^+^ ion signal at *m/z* 279.15491 (calcd for C_14_ H_24_ O_4_ Na, 279.15668). According to ^1^ H-, ^13^ C- and DEPT-NMR (Table 1), the compound contained two methyls, eight methylenes, one methane and one carbonyl. The ^1^ H−^1^ H COSY spectrum of compound **2** revealed three fragments (Figure 5) by the evident correlations of H-4/H-5, H-1′/H-2′/H-3′/H-4′ and H-1″/H-2″/H-3″/H-4″, respectively. The HMBC experiment (Figure 5) showed that the methine proton at *δ*_H_ 4.65 (H-4) is correlated with the carbons at *δ*_C_ 55.6 (C-6), 151.2 (C-3) and 202.7 (C-1); the methylene protons at *δ*_H_ 2.24 and 2.70 (H-4) are correlated with the carbons at *δ*_C_ 73.2 (C-4), 151.2 (C-3) and 202.7 (C-1); and the protons at *δ*_H_ 4.28 and 4.55 (H-6) are correlated with the carbons at *δ*_C_ 73.2 (C-4), 151.2 (C-3) and 153.6 (C-2). Those correlations established 2,4-dihydroxy-3-hydroxymethyl-cyclopent-2-en-1-one unit. Two straight-chain butyls are attached to 4-OH and 6-OH on the basis of the NOEs between H-6 to H-1″, and H-4 to H-1′ (Figure 5). The stereochemistry of 4-OH was determined by comparing the optical rotation values with (±)-4-hydroxy-3-methylcyclopent-2-enone (4 *S*-hydroxy-3-methyl-cyclopent-2-enone, $[\alpha {]}_{D}$, +3.36, *c* 0.375, CH_2_ Cl_2_; 4 *R*-hydroxy-3-methyl-cyclopent-2-enone, $[\alpha {]}_{D}$, − 4.7, *c* 0.35, CH_2_ Cl_2_) [25]. Therefore, compound **2** was determined as 4(*S*)-butoxy-3-(butoxymethyl)-2-hydroxycyclopent-2-en-1-one (Figure 4). This is a new compound.

The other compounds were identified as indole-3-carboxaldehyde (**3**) [26], 2,6-dimethoxy-1-anthracenecarboxaldehyde (**4**) [27], 7β,8β,11-triol-drimane (**5**) [28], 4-hydroxy-benzaldehyde (**6**), 4-hydroxybenzoic acid methyl ester (**7**), 4-hydroxy-benzoic acid (**8**), 4-hydroxy-benzeneacetic acid methyl ester (**9**), 4-hydroxy-benzeneacetic acid (**10**), ergosterol peroxide (**11**) [29], 3β,5α,9α-trihydroxy-ergosta-7,22-dien-6-one (**12**) [30] and adenosine (**13**) through a comparison of their experimental and reported spectroscopic data.

### 3.4. Nematicidal Activity of Compounds and Their Inhibition of Egg Hatching

The nematicidal activity of the isolated compounds against *M. incognita* up to a concentration of 400 μg mL^−1^ was determined. With the exception of 5-hydroxymethylfuran-2-carboxylic acid (**1**), none of the compounds displayed toxic effect on *M. incognita* at 400 μg mL^−1^. After being exposed to compound **1** for 24 h, the nematodes began to die and their body shape straightened. It caused significant mortality of *M. incognita* at 400, 200 and 100 μg mL^−1^ (Table 2). The nematicidal mortality attained 81.50% at 100 μg mL^−1^ for 48 h. The nematicidal effects varied with the concentration and exposure time, and the nematicidal activity differed significantly among the exposure times of 24 h, 48 h and 72 h at an identical concentration (Table 3). The mortality of *M. incognita* by abamectin attained 100% at 10 μg mL^−1^ for 24 h. 

The inhibition of egg hatching by 5-hydroxymethylfuran-2-carboxylic acid (**1**) was also assayed in our experiment. The other compounds were not tested further because only compound **1** showed nematicidal activity against *M. incognita*. The inhibition activity was tested in 96-well plates. The egg hatching of *M. incognita* was inhibited remarkably by compound **1** at 200 μg mL^−1^. The average number of hatched juveniles from one egg mass after three days of treatment with **1** was 11.17 at 200 µg mL^−1^, whereas the average number of hatched juveniles from one egg mass of the control treatment was 63.33 (Table 4). Hence, compound **1** can inhibit egg hatching of *M. incognita*.

## 4. Discussion

In the study, we isolated 13 metabolites from the EtOAc extract of *D. coniospora* YMF1.01759. Their structures displayed various compounds including polyketide, sesquiterpenoid, terpenes, nucleoside alkaloid and aromatic metabolites. Among these, 4(*S*)-butoxy-3-(butoxymethyl)-2-hydroxycyclopent-2-en-1-one (**2**) is a new natural product. The result shows that the strain can produce abundant metabolites.

An active assay demonstrated that 5-hydroxymethylfuran-2-carboxylic acid (**1**) had significant nematicidal activity and could affect the egg hatching of *M. incognita*. Compound **1** had been obtained from *Aspergillus* sp. earlier and showed effective nematicidal activities against the pine wood nematode *Bursaphelenchus xylophilus* and the free-living nematode *C. elegans* [31]. In our experiment, it showed activity against plant root-knot *M. incognita*. This indicated that the compound can kill different types of nematodes. Moreover, we observed it to display egg hatching inhibition activity, which had not been reported earlier. The compound 5-hydroxymethylfuran-2-carboxylic acid (**1**) was a furan, which belongs to polyketide. In previous studies, six furans were obtained from the fungus *Coprinus comatus*, which displayed nematicidal activity toward *Meloidogyne arenaria* and *P. redivivus* [32]. A nematicidal furan 5-(4-pentenyl)-2-furaldehyde was isolated from *Irpex lacteus*. It showed evident activity against *Aphelenchoides besseyi* [33]. This indicates that furans represent an important source for new natural nematicidal metabolites that may be developed as nematicidal agents.

As a typical endoparasitic fungus of nematodes, *D. coniospora* displayed strong pathogenic effect on nematodes by producing adhesive spores that attach to and penetrate the cuticle of nematodes. It exhibits certain potential for controlling plant-parasitic nematodes. This is the first report on the purification and identification of the main constituents of the species *D. coniospora*. The results indicate that metabolites have a synergistic effect with the pathogenic process of the ENF *D. coniospora* against nematodes. We demonstrated that *D. coniospora* YMF1.01759 could kill nematodes and inhibit egg hatching of *M. incognita* by producing spores or active metabolites. The biological control potential of the species *D. coniospora* against nematodes was expounded further.

## Figures and Tables

**Table 1:** The NMR Data of Compound **2** in CD_3_ OD (600 MHz).

Position	^1^ H	^13^ C	HMBC
1	-	202.7, s	-
2	-	153.6, s	-
3	-	151.2, s	-
4	4.65 (1 H, brd, *J* = 5.6 Hz)	73.2, d	C-1, C-1″, C-3, C-6
5	2.24 (1 H, brd, *J* =18.4 Hz)	41.8, t	C-1, C-3, C-4
	2.70 (1 H, dd, *J* =18.4, 5.7 Hz)		C-1, C-3
6	4.28 (1 H, d, *J* = 13.3 Hz)	55.6, t	C-2, C-3, C-4
	4.55 (1 H, d, *J* = 13.3 Hz)		C-2, C-3, C-4
1′	4.20 (2 H, m)	71.5, t	-
2′	1.58 (1 H, m)	33.2, t	-
	1.60 (1 H, m)		-
3′	1.44 (2 H, m)	20.4, t	C-4′
4′	0.93 (3 H, t, *J* = 7.4 Hz)	14.1, q	C-2′, C-3′
1″	3.55 (2 H, m)	70.7, t	C-2″
2″	1.44 (1 H, m)	33.1, t	C-1″, C-4″
	1.58 (1 H, m)		-
3″	0.96 (1 H, m)	19.9, t	-
	1.44 (1 H, m)		C-1″, C-4″
4″	0.94 (3 H, t, *J* = 7.4 Hz)	14.2, q	C-2″, C-3″

**Table 2:** Effect of Compound **1** on the Mortality (%) of *M. incognita* [mortality ± SD].

Concentration (µg mL^−1^)	24 h	48 h	72 h
400	90.10 ± 3.64 ^a^	99.51 ± 0.84 ^a^	100 ^a^
200	76.48 ± 3.86 ^a^	95.55 ± 1.7 ^a^	97.50 ± 0.91 ^a^
100	48.99 ± 2.05 ^a^	81.50 ± 2.46 ^a^	83.34 ± 1.67 ^a^
50	5.06 ± 0.87 ^a^	16.70 ± 0.82 ^a^	22.67 ± 2.37 ^a^
25	3.10 ± 0.49 ^a^	4.56 ± 0.59 ^a^	9.42 ± 0.69 ^a^
Control (3% methanol)	0.99 ± 0.08	2.03 ± 0.11	4.02 ± 0.14

^a^ *p* < 0.001.

**Table 3:** Variation in Percentage Mortality of *M. incognita* at each Concentration.

	400 µg mL^−1^	200 µg mL^−1^	100 µg mL^−1^	50 µg mL^−1^	25 µg mL^−1^
24 h−48 h	−7.3 ^a^	−19.5 ^a^	−33.5 ^a^	−3.8 ^b^	−1.5 ^b^
48 h−72 h	−0.4	−1.9	−1.3	−14.4 ^a^	−4.2 ^c^

^a^ *p* < 0.001; ^b^ *p* < 0.01; ^c^ *p* < 0.05.

**Table 4:** Inhibition of Egg hatching of *M. incognita* by compound **1**.

Concentration (µg mL^−1^)	Hatched Worms per Egg Mass ± SD		
	1 Day	2 Days	3 Days
200	5.83 ± 1.17 ^a^	8.17 ± 1.47 ^a^	11.17 ± 2.04 ^a^
100	12.00 ± 2.00 ^a^	18.50 ± 3.08 ^a^	24.00 ± 3.90 ^a^
50	13.50 ± 1.87 ^a^	35.17 ± 3.97 ^a^	44.00 ± 4.60 ^a^
Control (3% methanol)	37.33 ± 3.44	53.67 ± 3.33	63.33 ± 3.78

^a^ *p* < 0.001.

## Data Availability

Data sharing is not applicable to this article.
